# Conscience clause in emergency contraception: professional uncertainty, attitudes, and regulatory preferences among pharmacists and pharmacy students. A cross-sectional study

**DOI:** 10.3389/fphar.2026.1826694

**Published:** 2026-04-29

**Authors:** Justyna Czekajewska, Dariusz Walkowiak, Anna Jelińska, Jan Domaradzki

**Affiliations:** 1 Department of Social Sciences and Humanities, Poznan University of Medical Sciences, Poznan, Poland; 2 Department of Organization and Management in Health Care, Poznan University of Medical Sciences, Poznan, Poland; 3 Department of Pharmaceutical Chemistry, Poznan University of Medical Sciences, Poznan, Poland

**Keywords:** conscience clause, conscientious objection, emergency contraception, pharmacists, pharmacy practice, professional uncertainty, reproductive health policy

## Abstract

**Introduction:**

Access to emergency contraception (EC) and pharmacists’ professional autonomy remain subjects of legal and ethical debate in many healthcare systems. While pharmacists in Poland are increasingly involved in EC provision and pharmaceutical law emphasises the professional duty to dispense valid prescriptions, debate continues regarding pharmacists’ right to conscientious objection. However, unlike physicians, nurses, and midwives, pharmacists are not granted a statutory conscience clause (CC) under Polish law, and the possibility of invoking CC remains a matter of ongoing legal and ethical debate. This study aimed to assess community pharmacists’ and pharmacy students’ attitudes toward the CC in the context of EC provision.

**Methods:**

A cross-sectional survey was conducted among 381 respondents, including community pharmacists and fourth- and fifth-year pharmacy students. Data were collected using an anonymous, structured questionnaire that assessed attitudes toward EC, concerns about conscientious refusal to dispense EC, and preferences regarding potential legal regulation of the CC. Multivariable logistic regression analyses were used to identify factors associated with support for the CC and regulatory preferences.

**Results:**

More than half of respondents opposed allowing pharmacists to refuse EC dispensing on grounds of conscience clause (54.8%), while 33.8% supported such a right. Ethical concerns were reported more frequently when EC was intended for patients under 18 years of age. Legal concerns, particularly fear of civil liability, were reported more often than social or workplace consequences. Respondents strongly supported national-level ethical and legal regulations governing the use of the CC and generally opposed leaving such decisions to individual pharmacies. Higher religiosity was associated with greater support for the CC, whereas higher knowledge levels were associated with lower support.

**Conclusion:**

Attitudes toward the CC in the context of EC provision remain divided within the pharmacy community. The findings highlight the need for clear national regulatory frameworks and professional guidelines that reconcile pharmacists’ freedom of conscience with ensuring consistent patient access to time-sensitive reproductive health services.

## Introduction

Access to emergency contraception (EC) and the scope of pharmacists’ professional autonomy have become sources of regulatory and professional complexity in contemporary pharmacy practice. Regulatory changes introduced in Poland over the past decade concerning the availability of pharmacy-supplied emergency hormonal contraception have reignited discussions about the limits of pharmacists’ professional duties and the absence of an explicit statutory conscience clause (CC) for this professional group (Pawlikowski, 2019; Olszówka, 2019; Czekajewska et al., 2022a; Czekajewska et al., 2025). During the same period, access to EC in Poland has undergone systemic changes directly affecting pharmacy practice.

Debates surrounding pharmacists’ conscientious objection in the context of EC are not unique to Poland. In the United States and other jurisdictions, conflicts between access to EC and pharmacists’ moral or religious convictions have generated extensive legal, ethical, and policy discussions (Cantor and Baum, 2004; Card, 2007; Flynn, 2008; Kelleher, 2010). Reviews of state-level pharmacy regulations demonstrate substantial variability in how CC is recognised and operationalised, ranging from explicit protection clauses to mandatory referral or dispensing obligations (Achey and Robertson, 2022). Comparative analyses of pharmacists’ codes of ethics further reveal international differences in how professional autonomy, patient access, and moral integrity are balanced within regulatory frameworks (Wong et al., 2025a).

EC is a time-sensitive intervention whose effectiveness depends on prompt access and appropriate counselling (Rudzinski et al., 2023; Mierzejewska et al., 2024). Delays in obtaining the medication may reduce its preventive efficacy, making regulatory factors directly relevant to clinical outcomes. Poland remains one of the few European Union countries where EC requires a prescription, and proposals to allow over-the-counter sale continue to face political and social resistance (Ministerstwo Zdrowia, 2024; European Consortium for Emergency Contraception, 2026). Since the reintroduction of the prescription-only requirement in 2017, access has been repeatedly modified through legislative and executive measures (Ustawa o zmianie ustawy o świadczeniach opieki zdrowotnej finansowanych ze środków publicznych oraz niektórych innych ustaw, 2017).

A major shift occurred in 2024, when the Minister of Health introduced a pilot programme expanding pharmacists’ competencies in reproductive health (Rozporządzenie Ministra Zdrowia, 2024). Pharmacists are now authorised to conduct a structured interview, provide counselling, and issue a pharmaceutical prescription for ulipristal acetate to patients aged 15 years and older. EC provision has thus evolved from a dispensing activity into a structured clinical service delivered in community pharmacies.

Pharmacies are accessible healthcare facilities, and EC provision includes dispensing, counselling, and safeguarding patients’ rights, including privacy and informed decision-making (Susłowska et al., 2018). However, evaluations of EC services in Polish pharmacies revealed variability in counselling quality and limited reproductive health education, indicating gaps between regulatory expectations and practice (Susłowska et al., 2018). Recent analyses further show that regulatory shifts have not always translated into consistent implementation, and interpretative ambiguity regarding pharmacists’ competencies may undermine both patient access and professional confidence (Merks et al., 2025).

The expansion of pharmacists’ clinical role has not been accompanied by fully clarified legal and procedural standards, generating uncertainty in daily practice. A key issue concerns EC provision to minors. Although the pilot regulation allows access from the age of 15 (Rozporządzenie Ministra Zdrowia, 2024), legal analyses indicate that under the Act on Patients’ Rights (Act on Patients’ Rights and the Patients’ Rights Ombudsman, 2008), consent of a legal guardian may still be required for minors under 18 (Tomków et al., 2024). This inconsistency exposes pharmacists to potential civil, professional, or disciplinary liability.

Professional uncertainty is further reinforced by the broader legal framework governing pharmaceutical services (Pawlikowski, 2009). Pharmacists are obliged to ensure access to medicinal products and act in the patient’s best interest while complying with pharmaceutical law (Drozd, 2013; Ustawa Prawo farmaceutyczne, 2001). In this context, discussions on conscientious objection in healthcare emphasise the need to balance healthcare professionals’ freedom of conscience with patients’ right to access legally guaranteed services (Pawlikowski, 2024). Conflicts between professional duties and personal beliefs are therefore perceived as ethically challenging, particularly in relation to contraception (Piecuch et al., 2014; Czekajewska et al., 2022a; Czekajewska et al., 2026).

Importantly, the statutory CC in Poland applies to physicians, nurses, and midwives, but not to pharmacists (Czekajewska et al., 2022a; Olszówka, 2019). It is regulated in the Act on the Professions of Physician and Dentist (Ustawa o zawodach lekarza i lekarza dentysty, 1996, art. 39) and the Act on the Professions of Nurse and Midwife (Ustawa o zawodach pielęgniarki i położnej, 2011, Art. 12), whereas no analogous provision exists in pharmaceutical law (Drozd, 2013).

This regulatory asymmetry creates interpretative ambiguity. In the bioethical and legal literature, conscientious objection is understood as an individual moral conviction that leads a person to refuse to perform a specific professional or legal act. However, conscientious objection is not automatically legally protected, and its effectiveness depends on whether the applicable laws or professional regulations provide formal safeguards for such refusal (Pawlikowski, 2024). To prevent conflicts with patients’ rights and ensure effective access to healthcare services, it is essential to establish clear legal regulations and well-defined procedural guidelines. In the absence of such clear regulations, pharmacists may face uncertainty regarding dispensing decisions, documentation, and communication with patients, particularly in time-sensitive situations such as EC provision. Consequently, practices may vary across pharmacies, potentially leading to differences in patient access depending on individual interpretations of professional duties.

International analyses further indicate that refusal to dispense EC may be experienced by patients not merely as an inconvenience but as potential harm, particularly in time-sensitive contexts (McLeod, 2010). Previous studies suggest that pharmacists’ involvement in EC provision is shaped by tensions between professional identity, institutional expectations, and perceived moral responsibility (Maxwell et al., 2021). Furthermore, ethical and religious beliefs, along with misunderstandings about the mechanism of action of EC, have been shown to contribute to variability in dispensing practices, with decisions in some instances influenced by non-clinical factors such as the patient’s age or perceived moral deservingness (Cooper et al., 2008). At the same time, a systematic review indicates that community pharmacies are widely regarded as important access points for EC due to convenience, confidentiality, and the absence of appointment requirements; however, pharmacists also report concerns related to supply decisions, training needs, and professional responsibility, suggesting that variability in knowledge and confidence may affect the consistency and quality of service provision (Chirewa and Wakhisi, 2020). These findings underscore that the Polish debate forms part of a broader global discussion concerning the limits of professional autonomy and the safeguarding of equitable access to reproductive healthcare.

Previous Polish studies demonstrate that pharmacists’ attitudes toward EC are not uniform and are influenced by knowledge level, religiosity, and perceived legal constraints (Czekajewska et al., 2022b; Merks et al., 2025; Czekajewska et al., 2026). Although most pharmacists declare general support for EC use, uncertainty persists regarding counselling responsibilities, minors’ access, and professional discretion. This attitudinal variability, combined with regulatory ambiguity, may contribute to differences in service provision across community pharmacies.

The broader Polish debate on conscientious objection developed in the context of Resolution 1763 of the Parliamentary Assembly of the Council of Europe (2010), which affirmed the right to conscientious objection in lawful medical care (Council of Europe, 2010). This resolution influenced national legal interpretation and constitutional discourse on the scope of healthcare professionals’ freedom of conscience (Wyrok Trybunału Konstytucyjnego, 2015; Pawlikowski, 2019; Furtak-Niczyporuk, 2025). Empirical studies show that although most pharmacists have never refused to dispense on grounds of conscience, a minority would consider doing so if legally permitted (Piecuch et al., 2014), and many healthcare professionals perceive current regulations as unclear (Czekajewska et al., 2022b; Czekajewska et al., 2023). Public attitudes toward introducing a CC for pharmacists remain predominantly negative (Majchrowska et al., 2023).

Thus, in the context of EC, pharmacists operate at the intersection of expanding clinical responsibilities, incomplete regulatory guidance, and ethically sensitive services. From a clinical and health-systems perspective, the key issue concerns how regulations ensure patient access while maintaining professional legal security. Given the evolving regulatory landscape and emerging evidence of professional uncertainty, empirical data focusing specifically on community pharmacists and pharmacy students are needed.

This study, therefore, aimed to assess community pharmacists’ and pharmacy students’ attitudes toward the CC in the context of EC provision, including their concerns related to conscientious objection and their preferences regarding legal regulation, as well as to identify factors associated with these positions.

## Methods

### Study design

This study employed a cross-sectional survey design based on data collected through an anonymous, self-administered online questionnaire designed to analyse attitudes of pharmacists and pharmacy students toward the CC in the context of EC. The questionnaire assessed concerns related to refusal to dispense, preferences regarding legal regulation, and the identification of social and demographic factors significantly influencing respondents’ positions on this issue.

### Participants and setting

The study was conducted among fourth- and fifth-year full-time pharmacy students during the summer semester at the Poznan University of Medical Sciences (PUMS) and community pharmacists registered with the Wielkopolska Regional Chamber of Pharmacy in Poznan.

A total of 381 participants were included, comprising 223 students (132 of 143 fourth-year and 91 of 114 fifth-year students, corresponding to response rates of 92.3% and 79.8%, respectively; overall response rate: 86.8%) and 158 practicing pharmacists. At the time of the study, approximately 2,600 community pharmacists were practicing in the Wielkopolska region (Główny Urząd Statystyczny, 2025). The sample size was not determined using a formal calculation; instead, a convenience sampling approach was used. The final number of participants resulted from the availability and willingness of eligible individuals during the data collection period. Students were recruited during scheduled classes. Practicing pharmacists were recruited during four training sessions and conferences organized by the regional chamber during the study period.

The selection of participants was based on two considerations. Fourth- and fifth-year pharmacy students at PUMS already possess foundational and preclinical knowledge and are trained in analyzing patient cases, interpreting prescriptions, and providing pharmaceutical care. In contrast, licensed community pharmacists have full professional qualifications, practical experience, and legal authorization to practice and are registered in the national register of pharmacists.

Based on a systematic review of the literature, an original research instrument was developed in the form of a structured questionnaire. Its primary objective was to provide a comprehensive analysis of respondents’ attitudes toward various methods of contraception, with particular emphasis on EC. The study assessed concerns related to potential refusal to dispense prescriptions, preferences regarding legal regulations governing the application of the CC in the context of EC, and socio-demographic factors associated with respondents’ positions (Nowak and Walkowiak, 2023).

### Ethical issues

The study was performed in line with the principles of the Declaration of Helsinki (revised 2000) (Sawicka-Gutaj et al., 2022). Informed consent was obtained from all participants before completing the survey. Additionally, ethics approval and research governance approval were obtained from the PUMS Bioethics Committee (approval no. KB-197/25, dated 19 March 2025).

### Research tools

A structured questionnaire was developed based on a literature review of EC contraception and in collaboration with experts in pharmacy, public health, medical sociology, and bioethics. The questionnaire was elaborated according to the guidelines of the European Statistical System (Eurostat Brancato et al., 2005). A pilot version was tested among 35 third-year pharmacy students, who had already completed foundational and preclinical training (6th semester) but were not part of the target study population, which resulted in the reformulation of two questions. The final questionnaire consisted of four sections. The first section included questions regarding respondents’ attitudes toward the conscientious clause in the context of EC. The second section contained questions related to personal experiences and concerns associated with refusing to dispense EC due to conscientious objection. The third section focused on preferred legal and ethical solutions. The fourth section addressed demographic characteristics that significantly influence respondents’ ethical attitudes, levels of concern, and support for specific legal solutions.

All questions were closed-ended, with a defined set of clear response options. To ensure clarity and facilitate responses, specialized terminology was avoided. The question design allowed respondents to express their degree of agreement on a five-point Likert scale, from “Strongly disagree” to “Strongly agree,” allowing participants to express their views clearly. Most questions also included a neutral option: “I do not know”.

To assess religiosity, emphasizing the subjective impact of religious beliefs on daily life and community relationships, the validated Polish adaptation of the Religious Commitment Inventory-10 (RCI-10-PL) by Worthington et al. was used. This 10-item scale measures the extent of religious commitment, with higher scores indicating greater religiosity. In this study, the RCI-10-PL demonstrated high reliability (Cronbach’s α = 0.948, 95% CI: 0.938–0.957), and principal component analysis confirmed a satisfactory single-factor model fit.

### Data collection

The study was conducted between March 2025 and June 2025 among pharmacy students at PUMS and practicing pharmacists. Using a non-probability (convenience-based) sampling approach, questionnaires were distributed during regular classes for fourth- and fifth-year full-time students and during training sessions and conferences organized by Wielkopolska Regional Chamber of Pharmacy in Poznan for pharmacists. All eligible individuals present at the time of data collection were invited to participate, reducing selective inclusion. Before participation, all participants were informed about the purpose of the project and provided consent. All respondents received a QR code, which, when scanned with their smartphones, gave them access to the online questionnaire. The principal investigator (JC) was present throughout data collection to address possible questions. Participation was voluntary and anonymous, which may have reduced social desirability bias. The questionnaire was completed individually via an online platform without instructor supervision, ensuring independence of responses and minimizing potential power imbalance. Completing the questionnaire took on average 10–15 min.

### Data analysis

Descriptive statistics were calculated for all participant characteristics and survey responses. Categorical variables are presented as counts and percentages, and continuous variables are reported as means, standard deviations, medians, and ranges (Tables 1, 2). Logistic regression analyses were performed to assess associations between respondent characteristics (age, sex, occupational status, religiosity [RCI-10], Knowledge Index, ideological orientation, and political preferences) and: (i) support for the CC, (ii) concerns about refusing to dispense EC, and (iii) preferences regarding legal regulations of the CC (Tables 5, 6). For analyses concerning respondents’ views on access to EC, separate models were constructed for scenarios referring to individuals aged <18 years and ≥18 years, where appropriate. All regression models report odds ratios (OR) with 95% confidence intervals (CI). Model fit was assessed using Nagelkerke R^2^, and statistical significance was defined as p < 0.05. All analyses were conducted using JASP 0.95.4.

**TABLE 1:** Participants characteristics.

Characteristics	Total	
	(n = 381)	%
*Gender*		
female	272	71.4
male	109	28.6
*Occupational status*		
student	223	58.5
pharmacist	158	41.5
*Domicile*		
up to 10,000 inhabitants	62	16.3
10-50,000 inhabitants	41	10.8
51-100,000 inhabitants	19	5
101-500,000 inhabitants	69	18.1
above 500,000 inhabitants	190	49.9
*Age (in years)*		
Range	21–64	
Mean (95% CI)	30.1 (29–31.2)	
SD	10.8	
Median	24	
*Religious Commitment Inventory-10*		
Range	10–50	
Mean (95% CI)	22.7(21.6–23.8)	
SD	10.6	
Median	21.5	
*Knowledge Index*		
Range	−2.0 – 20.0	
Mean (95% CI)	12.8(12.3–13.2)	
SD	4.8	
Median	14.0	
*How would you describe your position on ideological (customary, ethical, moral) issues?*		
strongly liberal	94	24.7
rather liberal	139	36.5
centrist	101	26.5
rather conservative	31	8.1
strongly conservative	16	4.2
*How would you describe your political preferences on the left - right spectrum?*		
strongly left-wing	57	15
rather left-wing	113	29.7
center	151	39.6
rather right-wing	41	10.8
strongly right-wing	19	5

**TABLE 2:** Personal experiences and concerns of community pharmacists and pharmacy students regarding the conscience clause (CC) in the context of emergency contraception (EC).

Concerns related to the conscience clause in the context of emergency contraception	Definitely not n (%)	Rather not n (%)	I do not know n (%)	Rather yes n (%)	Definitely yes n (%)
*Should a pharmacist be allowed to invoke a CC to refuse dispensing EC?*	140 (36.7)	69 (18.1)	43 (11.3)	63 (16.5)	66 (17.3)
*Would you have any concerns about refusing to fill a prescription for EC?*	75 (19.7)	109 (28.6)	84 (22)	93 (24.4)	20 (5.2)
*What concerns would you have about invoking the CC regarding EC?*					
*Fear of losing the job*	109 (28.6)	112 (29.4)	91 (23.9)	57 (15)	12 (3.2)
*Fear that relationships with colleagues will deteriorate and become tense and conflict-ridden*	103 (27)	93 (24.4)	88 (23.1)	84 (22)	13 (3.4)
*Fear of not receiving a professional promotion*	109 (28.6)	121 (31.8)	93 (24.4)	50 (13.1)	8 (2.1)
*Fear that it will result in negative opinions/gossip about me*	103 (27)	84 (22)	84 (22)	97 (25.5)	13 (3.4)
*Fear that it will lead to the denial of support from colleagues in difficult situations*	104 (27.3)	97 (25.5)	90 (23.6)	77 (20.2)	13 (3.4)
*Fear of being treated with contempt and disregard by colleagues and patients*	110 (28.9)	92 (24.1)	86 (22.6)	81 (21.3)	12 (3.2)
*Fear that colleagues will withhold important information (e.g., amendments to the regulations on issuing and dispensing prescriptions)*	120 (31.5)	86 (22.6)	100 (26.5)	63 (16.5)	12 (3.2)
*Fear of disciplinary proceedings*	112 (29.4)	92 (24.1)	87 (22.8)	69 (18.1)	21 (5.5)
*Fear of a potential civil lawsuit from the patient (pharmacy customer) or their family*	86 (22.6)	70 (18.4)	96 (25.2)	95 (24.9)	34 (8.9)
*I would have no concerns*	67 (17.6)	88 (23.1)	84 (22)	71 (18.6)	71 (18.6)

## Results

The study included 381 respondents (71.4% female), of whom 58.5% were students and 41.5% practicing pharmacists (Table 1). Nearly half (49.9%) lived in cities with over 500,000 inhabitants. Participants were aged 21–64 years (mean 30.1 ± 10.8). Religious commitment, assessed using the RCI-10, ranged from 10 to 50, with a mean score of 22.7 ± 10.6. The Knowledge Index ranged from −2.0 to 20.0, with a mean score of 12.8 ± 4.8 (Czekajewska et al., 2026). While most respondents identified as liberal (61.2%), while 26.5% were centrist, and 12.3% conservative; politically, 44.7% declared left-wing views, 39.6% centrist, and 15.8% right-wing.

Table 2 presents respondents’ attitudes toward invoking the CC in the context of EC and related concerns. The majority of respondents (54.8%) opposed granting pharmacists the right to invoke a CC to refuse dispensing EC, while 33.8% expressed support for such a right. Nearly half (48.3%) declared no concerns about refusing to fill a prescription, while 29.6% reported potential concerns.

Across specific items, anticipated legal consequences were the most frequently endorsed concern, particularly fear of a civil lawsuit (33.8%). Social repercussions, such as negative opinions or gossip (28.9%) and deterioration of collegial relationships (25.4%), were reported less often. Concerns related to formal employment consequences were less frequently indicated (disciplinary proceedings: 23.6%; job loss: 18.2%; lack of promotion: 15.2%). Approximately one-quarter of respondents selected “I do not know” across items, indicating substantial uncertainty regarding the potential implications of invoking the CC.

Table 3 presents ethical objections to selected contraceptive methods according to the age of the potential user (>18 vs. <18 years). Differences by age were statistically significant for all methods (all p < .001). For users over 18 years of age, the vast majority of respondents reported no ethical objections to any method (ranging from 84.8% to 94.2%).

**TABLE 3:** Ethical attitudes of community pharmacists and pharmacy students toward selected contraceptive methods.

Ethical attitudes toward selected contraceptive methods	Age of the person to use a specific contraception	Definitely not n (%)	Rather not n (%)	I do not know n (%)	Rather yes n (%)	Definitely yes n (%)	p
*Which types of contraception provoke your ethical objections*							
Hormonal contraceptives	>18 years	276 (72.4)	75 (19.7)	8 (2.1)	18 (4.7)	4 (1.1)	<.001
	<18 years	156 (40.9)	119 (31.1)	26 (6.8)	51 (13.4)	29 (7.6)	
Mechanical methods of contraception, e.g., vaginal rings, condoms	>18 years	295 (77.4)	64 (16.8)	5 (1.3)	13 (3.4)	4 (1.1)	<.001
	<18 years	210 (55.1)	111 (29.1)	13 (3.4)	26 (6.8)	21 (5.5)	
Chemical barrier methods, e.g., globules, foams, creams, spermicidal fluids	>18 years	279 (73.2)	68 (17.8)	12 (3.2)	14 (3.7)	8 (2.1)	<.001
	<18 years	112 (29.4)	82 (21.5)	56 (14.7)	78 (20.5)	53 (13.9)	
Methods that prevent fertilisation, e.g., IUD	>18 years	265 (69.6)	70 (18.4)	9 (2.4)	23 (6)	14 (3.7)	<.001
	<18 years	122 (32)	100 (26.2)	35 (9.2)	75 (19.7)	49 (12.9)	
Postcoital contraception (the “morning-after pill”)	>18 years	249 (65.4)	74 (19.4)	13 (3.4)	29 (7.6)	16 (4.2)	<.001
	<18 years	122 (32)	100 (26.2)	35 (9.2)	75 (19.7)	49 (12.9)	

In contrast, for users under 18 years of age, the proportion reporting no ethical objections decreased across all methods (ranging from 50.9% to 84.2%), accompanied by a corresponding increase in expressed ethical concerns (12.3%–34.4%). This shift was most pronounced for chemical barrier methods, intrauterine devices, and postcoital contraception, where approximately one-third of respondents reported objections.

Respondents showed strong support for formal regulatory approaches to the CC in EC (Table 4). The majority favored national regulations developed by pharmaceutical authorities (74.0%) and the introduction of specific ethical and legal regulations defining the scope, conditions, and procedures for pharmaceutical services, with mandatory compliance (76.9%). In contrast, most respondents opposed delegating decisions to pharmacy managers in cases of refusal (60.1%). A complete prohibition of the CC was rejected by 49.9% of respondents and supported by 27.0%, while 23.1% were undecided.

**TABLE 4:** Preferred legal regulations concerning the conscience clause (CC) in emergency contraception (EC).

Regulation approach	Definitely not n (%)	Rather not n (%)	I do not know n (%)	Rather yes n (%)	Definitely yes n (%)
*National regulations developed by pharmaceutical authorities (e.g., the Main Pharmaceutical Inspectorate or the Supreme Pharmaceutical Chamber)*	14 (3.7)	33 (8.7)	52 (13.6)	138 (36.2)	144 (37.8)
*Introduction of specific ethical and legal regulations defining the scope, conditions, and procedures for pharmaceutical services, with mandatory compliance*	23 (6)	20 (5.2)	45 (11.8)	134 (35.2)	159 (41.7)
*Decisions made by the pharmacy manager in situations where an employee refuses to provide a pharmaceutical service*	91(23.9)	138 (36.2)	92 (24.1)	48 (12.6)	12 (3.2)
*Complete prohibition of the CC for pharmacists concerning EC*	101 (26.5)	89 (23.4)	88 (23.1)	47 (12.3)	56 (14.7)

Logistic regression analyses examined predictors of support for the CC and concerns about refusing to dispense EC (Table 5). Higher religiosity (RCI-10) was consistently associated with greater support for the CC (OR = 1.055, 95% CI: 1.021–1.089, p < 0.001), whereas higher Knowledge Index scores were associated with lower support (OR = 0.870, 95% CI: 0.810–0.934, p < 0.001).

**TABLE 5:** Logistic regression parameters for the conscience clause (CC) in emergency contraception (EC), also with respect to pharmacy patients’ age.

Regression parameters	*Should a pharmacist be allowed to invoke a CC to refuse dispensing EC?*	*Would you have any concerns about refusing to fill a prescription for EC?*	*EC (“morning after pill”)* >18 years	*EC (“morning after pill”)* <18 years
	OR (95% CI)	OR (95% CI)	OR (95% CI)	OR (95% CI)
Intercept	6.963 (0.697; 69.552)	0.241*** (0.135; 0.430)	7.117 × 10^−10^ (0; ∞ )	0.253* (0.066; 0.967)
Age	0.909** (0.866; 0.955)	n.s.	n.s.	​
Knowledge index	0.870*** (0.810; 0.934)	n.s.	n.s.	0.921* (0.861; 0.985)
RCI-10	1.055*** (1.021; 1.089)	1.041*** (1.018; 1.064)	1.081*** (1.031; 1.134)	1.088*** (1.054; 1.122)
Men vs. Women	n.s.	n.s.	n.s.	0.415* (0.212; 0.810)
Students vs. pharmacists	0.243** (0.096; 0.614)	n.s.	n.s.	0.436* (0.229; 0.829)
Political orientation: Strongly left-wing vs. rather left-wing	4.260* (1.382; 13.132)	n.s.	n.s.	n.s.
Political orientation: Strongly left-wing vs. centrist	7.349*** (2.500; 21.602)	n.s.	n.s.	n.s.
Political orientation: Strongly left-wing vs. rather right-wing	15.366*** (4.063; 58.110)	n.s.	n.s.	n.s.
Political orientation: Strongly left-wing vs. strongly right-wing	n.s.	n.s.	n.s.	n.s.
Worldview: Strongly liberal vs. rather liberal	n.s.	n.s.	n.s.	n.s.
Worldview: Strongly liberal vs. centrist	n.s.	n.s.	n.s.	n.s.
Worldview: Strongly liberal vs. rather conservative	n.s.	n.s.	n.s.	18.585*** (4.322; 79.912)
Worldview: Strongly liberal vs. strongly conservative	n.s.	n.s.	n.s.	n.s.
R^2^ Nagelkerke	0.460	0.059	0.569	0.448
p-value for model	0.002	<0.001	<0.001	0.015

* p-value <0.05; ** p-value <0.01; *** p-value <0.001; n.s. not significant.

Support decreased with increasing age (OR = 0.909, 95% CI: 0.866–0.955, p < 0.01), and students were less likely than practicing pharmacists to indicate support (OR = 0.243, 95% CI: 0.096–0.614, p < 0.01). Ideological orientation also played a role, with strongly left-wing political identification and a strongly liberal worldview associated with opposition (ORs up to 18.585).

Higher religiosity was likewise associated with greater concerns about refusing to dispense EC (OR = 1.041, 95% CI: 1.018–1.064, p < 0.001), while students and male respondents were less likely to report such concerns. In analyses stratified by patient age, religiosity remained a significant positive predictor of support in both age groups, whereas higher knowledge was associated with fewer concerns for patients under 18 years of age. Model fit ranged from Nagelkerke R^2^ = 0.059 to 0.569, and all models were statistically significant (p = 0.002–<0.001).

Logistic regression analyses examined predictors of preferences regarding legal regulations of the CC in EC (Table 6). Support for national agreements developed by pharmaceutical authorities was negatively associated with respondent age (OR = 0.948, 95% CI: 0.921–0.976, p < 0.001) and positively associated with religiosity (RCI-10; OR = 1.041, 95% CI: 1.000–1.084, p < 0.05). Respondents identifying as strongly left-wing (vs. rather right-wing) had substantially lower odds of support (OR = 0.081, 95% CI: 0.019–0.349, p < 0.001; Nagelkerke R^2^ = 0.122, p = 0.044). Support for mandatory ethical and legal regulations with obligatory compliance decreased with age (OR = 0.949, 95% CI: 0.917–0.982, p < 0.01) and increased with religiosity (RCI-10; OR = 1.107, 95% CI: 1.055–1.163, p < 0.001) and Knowledge Index (OR = 1.095, 95% CI: 1.017–1.178, p < 0.05; Nagelkerke R^2^ = 0.177, p = 0.016). No predictors reached statistical significance for preferences regarding decisions made solely by the pharmacy manager.

**TABLE 6:** Logistic regression parameters for preferences on legal regulations of the conscience clause (CC) related to emergency contraception (EC).

Regression parameters	Through national agreements made exclusively by pharmaceutical experts, e.g., the Main Pharmaceutical Inspectorate or the Supreme Pharmaceutical Chamber	By introducing specific ethical and legal regulations defining the scope, conditions, and pharmaceutical services, and obliging employees to comply with them	Through decisions made solely by the manager of the pharmacy, where an employee refused to provide a pharmaceutical service	By completely prohibiting the CC among pharmacists regarding EC
	OR (95% CI)	OR (95% CI)	OR (95% CI)	OR (95% CI)
Intercept	26.043*** (5.959; 113.813)	1.282 (0.326; 5.036)	n.s.	1.235 (0.786; 1.942)
Age	0.948*** (0.921; 0.976)	0.949** (0.917; 0.982)	n.s.	n.s.
Knowledge index	n.s.	1.095* (1.017; 1.178)	n.s.	n.s.
RCI-10	1.041* (1.000; 1.084	1.107*** (1.055; 1.163)	n.s.	n.s.
Men vs. women	n.s.	n.s.	n.s.	n.s.
Students vs. pharmacists	n.s.	n.s.	n.s.	n.s.
Political orientation: Strongly left-wing vs. rather left-wing	n.s.	n.s.	n.s.	n.s.
Political orientation: Strongly left-wing vs. centrist	n.s.	n.s.	n.s.	n.s.
Political orientation: Strongly left-wing vs. rather right-wing	0.081*** (0.019; 0.349)	n.s.	n.s.	n.s.
Political orientation: Strongly left-wing vs. strongly right-wing	n.s.	n.s.	n.s.	n.s.
Worldview: Strongly liberal vs. rather liberal	n.s.	n.s.	n.s.	0.544* (0.300; 0.986)
Worldview: Strongly liberal vs. centrist	n.s.	n.s.	n.s.	0.270*** (0.133; 0.549)
Worldview: Strongly liberal vs. rather conservative	n.s.	0.158* (0.035; 0.718)	n.s.	n.s.
Worldview: Strongly liberal vs. strongly conservative	n.s.	n.s.	n.s.	n.s.
R^2^ Nagelkerke	0.122	0.177	​	0.227
p-value for model	0.044	0.016	n.s.	<0.001

* p-value <0.05; ** p-value <0.01; *** p-value <0.001; n.s. not significant.

Support for the complete prohibition of the CC among pharmacists was associated exclusively with worldview orientation. Compared with respondents identifying as strongly liberal, those identifying as rather liberal (OR = 0.544, 95% CI: 0.300–0.986, p < 0.05) and centrist (OR = 0.270, 95% CI: 0.133–0.549, p < 0.001) had significantly lower odds of supporting prohibition. No significant associations were observed for age, religiosity, knowledge, gender, or professional status.

## Discussion

This study analysed attitudes toward the regulation of the CC in the context of EC. It revealed a lack of consensus within the pharmacy community, indicating a division of opinions that may translate into professional practice and ethical conflicts. The findings showed that the greatest concerns were related to dispensing EC to individuals under 18 years of age. At the same time, the lack of guaranteed access to this service also raised concerns, primarily regarding legal rather than social or professional consequences. To balance patients’ rights and the protection of minors with the moral and legal duties of healthcare personnel, pharmacists favored precise, systemic, and nationwide ethical and legal regulations over arbitrary solutions at the pharmacy level. Support for institutional or expert-based regulation was shaped not only by professional knowledge but also by broader value systems. Importantly, religiosity and political self-identification were more consistently associated with preferences than professional status, suggesting that the debate over EC extends beyond professional ethics and into moral identity and personal worldview.

In Poland, opinions on over-the-counter access to EC have remained polarized for more than a decade. Data from the Polish Opinion Research Center showed that public support and opposition were almost evenly divided (43% vs. 44%), with many respondents perceiving the EC as harmful to women’s health or equivalent to abortion (Centrum Badania Opinii Społecznej, 2015). More recent data confirm the persistence of this divide: 50% of respondents support over-the-counter access for individuals aged 15 and above, while 42% oppose it, particularly among those with right-wing political views, higher religiosity, and lower educational levels (Centrum Badania Opinii Społecznej, 2024). Public attitudes toward conscience-based refusal in reproductive healthcare also appear restrictive: a nationwide survey indicated that 76% of respondents opposed allowing pharmacists to refuse dispensing contraceptives on grounds of conscience (Centrum Badania Opinii Społecznej, 2014). Similar divisions are observed within the pharmacy community. Although most pharmacists and pharmacy students support the use of EC (84.5%), opinions remain split regarding its over-the-counter availability (40.2% supporting vs. 52.9% opposing) and provision to minors without parental consent (40.8% vs. 48.9%) (Czekajewska et al., 2026). The presented data are consistent with the findings of this study, as shown in Table 2, where opinions regarding pharmacists’ right to invoke the CC are divided, reflecting varied respondent perspectives (33.8% vs. 54.8%).

The first key finding is that variation in attitudes within the pharmacy community toward EC may influence the legal interpretation and practical application of the CC, potentially affecting access to services for individuals under 18 years of age. This observation is consistent with previous analyses of regulatory ambiguity and professional uncertainty in the provision of EC in Poland (Czekajewska et al., 2022b; Czekajewska et al., 2023; Merks et al., 2025; Susłowska et al., 2018). Similar patterns have been reported internationally. A scoping review by Nona et al. indicated that pharmacists do not consistently demonstrate supportive attitudes toward dispensing EC to minors (Nona et al., 2025). Likewise, Hussainy et al. found that pharmacists with limited knowledge of existing guidelines were more likely to refuse dispensing EC in situations lacking parental consent or a prescription (Hussainy et al., 2011). Other studies suggest that restrictions in EC provision are often shaped less by clinical contraindications and more by socio-cultural or religious norms regarding adolescent sexuality (Apikoglu-Rabus et al., 2012; Belachew et al., 2017; Mody et al., 2019; Akande-Sholabi et al., 2023; Shaukat and Al-Metwali, 2022).

Comparable attitudes have also been observed among paediatric healthcare professionals. Nurses, more often than physicians, expressed reservations about providing EC to adolescents, particularly in cases involving voluntary sexual activity, reflecting concerns about adolescents’ maturity and responsibility in sexual decision-making (Miller et al., 2011). These findings suggest that professional attitudes toward EC provision to minors are shaped not only by legal regulations but also by broader ethical interpretations of professional responsibility within healthcare systems (Pawlikowski, 2019; Pawlikowski, 2009).

The second key finding indicates that ethical concerns are concentrated around specific reproductive health products rather than distributed evenly across all contraceptive methods. As shown in Table 3, representatives of the pharmaceutical community more frequently reported ethical objections to chemical methods, intrauterine devices (IUDs) and postcoital contraception, which is consistent with previous studies among Polish healthcare professionals showing that contraceptive services are among the most common contexts in which the CC is considered (Czekajewska et al., 2022b; Piecuch et al., 2014; Majchrowska et al., 2023).

Comparable trends have also been reported internationally. A study among Slovak pharmacists found that 43.5% considered the CC an acceptable basis for refusing EC dispensing, and nearly one-third reported exercising this right in practice (Koláfi et al., 2016). Research from the United Kingdom and the United States likewise indicates that EC is among the pharmaceutical services most commonly associated with conscientious objection, with religiosity emerging as a significant determinant of refusal decisions. This, in turn, may result in tension between patients’ right to access medication and pharmacists’ moral integrity (Maxwell et al., 2021; Davidson et al., 2010).

International literature indicates that barriers to obtaining EC in pharmacy settings may have consequences beyond inconvenience, given the time-sensitive nature of the intervention (McLeod, 2010). At the same time, dispensing practices may vary due to tensions between professional duties, institutional expectations, and pharmacists’ moral beliefs, as well as misunderstandings regarding the mechanism of action of EC (Cooper et al., 2008; Maxwell et al., 2021). Community pharmacies are widely recognised as key access points for timely EC services, yet pharmacists report uncertainties related to counselling, training, and professional responsibility that may affect service consistency (Chirewa and Wakhisi, 2020).

Against this background, the situation in Poland appears consistent with broader international patterns linking religiosity to attitudes toward the CC. Previous research shows that healthcare professionals most frequently associate the clause with abortion, followed by contraception-related services (Czekajewska et al., 2022b). Legal analyses of the Polish healthcare system also emphasize that the absence of an explicit CC for pharmacists creates interpretative ambiguity regarding the scope of professional duties (Drozd, 2013; Olszówka, 2019). At the same time, religiosity strongly influences these attitudes, with more religious professionals significantly more likely to support the application of the clause in reproductive healthcare contexts (Czekajewska et al., 2023). However, moral acceptance of conscientious objection does not necessarily translate into its practical use. While 26% of religious healthcare professionals reported an intention to invoke the clause, only 13% declared having actually done so in practice (Czekajewska et al., 2023). This discrepancy suggests that healthcare professionals often experience tension between personal beliefs and professional responsibilities. At the same time, empirical studies indicate that the exercise of the CC may lead to practical consequences for healthcare access. Refusal to provide legally permitted reproductive health services has been shown to delay care, shift responsibility onto patients to seek alternative providers, and create organisational barriers within healthcare systems (Davis et al., 2022; Krawutschke et al., 2024; Pawlikowski, 2024).

The third key finding is that legal and professional concerns related to invoking the CC elicit greater apprehension among pharmacists than potential social repercussions or workplace reactions. As shown in Table 2, pharmacists more frequently reported fear of legal consequences and civil liability than concerns related to social opinion or professional relationships. A similar pattern has been observed among nurses and midwives, where the risk of legal claims from patients or their families was the primary source of concern: 33.8% of respondents indicated fear of potential civil lawsuits, whereas worries about social consequences, such as workplace gossip or deterioration of professional relationships, were reported less frequently (Czekajewska et al., 2025).

Previous research also demonstrates that healthcare professionals frequently perceive current regulations governing the CC as unclear or difficult to interpret (Czekajewska et al., 2022b). Indeed, large proportions of healthcare professionals report uncertainty regarding the legal conditions for invoking the clause and the obligation to ensure patients’ access to services, indicating a need for clearer regulatory frameworks (Czekajewska et al., 2022b; Czekajewska et al., 2025). This uncertainty reflects broader debates in Polish legal scholarship regarding the balance between healthcare professionals’ freedom of conscience and patients’ rights within the healthcare system (Furtak-Niczyporuk, 2025; Pawlikowski, 2019; Pawlikowski, 2024). These concerns are well-founded, as previous analyses indicate that refusal to perform legally permitted procedures may result in delays in care, transfer of responsibility to patients seeking alternative providers, and potential limitations in access to healthcare services (Davis et al., 2022; Krawutschke et al., 2024). In the context of pharmaceutical services, such risks may be particularly relevant because community pharmacies are often the first and most accessible point of contact within the healthcare system (Susłowska et al., 2018; Merks et al., 2025).

The fourth key finding suggests that pharmacists and pharmacy students more often favor the development of clear national guidelines rather than the complete prohibition of the CC. As shown in Table 4, nearly half of pharmacists did not support a complete ban on invoking the CC in the context of providing EC, suggesting a preference for clarifying regulations rather than eliminating them. Similar conclusions have been drawn in international literature, which shows that pharmacists frequently attempt to reconcile personal moral values with professional obligations toward patients (Wong et al., 2025b). Studies also demonstrate that pharmacists’ decisions regarding conscientious objection depend on contextual factors such as workplace environment, institutional expectations, and professional norms (Richman et al., 2012; Piecuch et al., 2014; Lee et al., 2015; Maxwell et al., 2021). Evidence from qualitative and survey studies further suggests that pharmacists often attempt to balance personal beliefs with professional duties. In morally conflicting situations, some pharmacists report prioritising professional responsibilities over personal religious convictions, while others emphasise that refusal may be difficult when a medication has been legally prescribed by a physician (Cooper et al., 2008; Gülpınar et al., 2021). These findings highlight the importance of clear ethical and legal frameworks to reduce arbitrariness and prevent workplace conflicts.

These findings align with Polish studies indicating that pharmacists’ attitudes toward CC are shaped by ethical reasoning, perceived legal constraints, and professional identity (Czekajewska et al., 2022b; Majchrowska et al., 2023).

Finally, the study confirms that religiosity, political views, and personal worldview are key determinants of pharmacists’ and pharmacy students’ attitudes toward the CC in the context of EC. More religious individuals are more likely to accept the use of the clause and consider invoking it in situations of moral conflict, while simultaneously expressing greater concerns about potential personal consequences (Czekajewska et al., 2023). This finding reflects the broader nature of the debate, which extends beyond professional ethics and legal regulation to encompass moral identity and personal belief systems. Similar associations between religiosity and attitudes toward EC provision have been documented in the United Kingdom and the United States (Maxwell et al., 2021; Davidson et al., 2010). Evidence from several Western European countries further demonstrates that it is possible to reconcile the protection of healthcare professionals’ moral integrity with ensuring patient access to legal services, provided that regulatory frameworks clearly define the procedures for invoking conscientious objection and ensure effective referral mechanisms (Chavkin et al., 2017).

The analysis of odds ratios (OR) further illustrates the practical significance of predictors related to attitudes toward the CC and respondents’ preferences regarding its legal regulation, as presented in Tables 5, 6. Higher religiosity (RCI-10; OR = 1.055) indicates that each additional point on this scale is associated with a a 5.5% increase in the odds of supporting the CC, reflecting a moderately positive relationship. In contrast, a higher Knowledge Index score (OR = 0.870) corresponds to an approximately 13% decrease in the odds of support for the clause per additional point, indicating a negative association. Support for the CC also declines with age (OR = 0.909), meaning that with each additional year of life, the odds of support decrease by about 9%, reflecting a slight negative association. The study also highlighted the significant role of ideological orientation, with strong left-wing political identification and a distinctly liberal worldview associated with opposition to the CC, with odds ratios of 18.6. These findings emphasize that both personal factors (religiosity, worldview) and professional factors (knowledge, experience) influence attitudes toward the CC and support for formal mandatory regulations, underscoring the importance of clear regulatory frameworks that reconcile pharmacists’ moral integrity with ensuring patients’ access to legally permitted services.

### Limitations

Several limitations should be considered when interpreting the findings. First, the cross-sectional design precludes causal inference. Therefore, it cannot be determined whether, for example, higher religiosity leads to greater support for the CC, but only that these variables are associated. Future longitudinal or prospective studies could help clarify causal relationships and provide insight into how such attitudes evolve in response to professional experience, policy changes, or educational interventions. Second, the study relied on self-reported data, which may be influenced by social desirability bias, particularly given the ethically sensitive nature of EC and the CC. The use of a web-based questionnaire may also have introduced self-selection effects, as individuals with stronger views on the topic could have been more inclined to participate. For instance, individuals with strong opinions about EC may have been more likely to participate, potentially skewing the results toward more polarized views. In addition, a convenience sampling approach was used, involving pharmacy students from a single university and pharmacists affiliated with one regional chamber, which may limit the generalizability of the findings. As a result, the findings may not fully reflect the views of all pharmacists in Poland. Future research could employ probability-based sampling methods and include larger, more diverse, and preferably nationally representative samples from multiple institutions and regions to improve representativeness and enhance external validity. Third, ideological and worldview variables were operationalized using categorical self-identification. While analytically necessary, this approach may oversimplify complex value systems and potentially mask within-category heterogeneity. Consequently, subtle differences within ideological groups may not have been captured. Additionally, the survey did not capture certain contextual factors, such as workplace characteristics, prior training related to EC, or organizational environment, which might further explain observed differences. The omission of these variables may have resulted in residual confounding. Future studies could incorporate more nuanced measurement scales and include additional contextual and organizational variables to better capture these complexities. Fourth, some regression coefficients were estimated with limited precision. In particular, certain ideological categories (e.g., strongly conservative worldview) were associated with very large standard errors and extremely wide confidence intervals, in some cases approaching non-informative bounds. This suggests sparse data in these subgroups and potential quasi-complete separation, limiting the stability and interpretability of these estimates. Therefore, these estimates should be interpreted with caution, as they may not reliably reflect true associations. Increasing sample sizes in underrepresented subgroups in future studies may improve the precision and stability of these estimates. Fifth, although multiple predictors were included in the models, the explained variance was modest (Nagelkerke R^2^ ranging from 0.122 to 0.227), indicating that additional relevant factors influencing attitudes toward the CC may not have been captured. Future research should explore additional psychological, educational, and professional variables to enhance the explanatory power of the models. Sixth, some models yielded no statistically significant predictors, suggesting either limited statistical power for certain outcomes or genuinely weak associations. The absence of significant effects in these models should be interpreted cautiously, particularly given possible subgroup imbalances. Future studies with larger samples or alternative modeling approaches may help to better identify relevant predictors. Finally, the findings should be interpreted within the specific legal and cultural context in which the study was conducted, as national regulatory frameworks and sociocultural factors may shape attitudes differently across healthcare systems. Consequently, the transferability of the results to other countries may be limited. Comparative cross-national studies could help assess the generalizability of these findings.

Despite these limitations, the study also has several strengths. It addresses an underexplored area at the intersection of pharmacy practice, ethics, and health policy, providing empirical data on attitudes toward the CC in the context of EC. By including both practicing pharmacists and pharmacy students, the study captures perspectives from current and future professionals, allowing insight into potential generational differences. The analysis incorporated multiple explanatory variables, including religiosity, knowledge, and sociodemographic characteristics, enabling a multidimensional examination of factors associated with regulatory preferences. Finally, the use of multivariable modelling allowed for the assessment of independent associations between these factors and respondents’ views, contributing to a more nuanced understanding of the issue.

## Conclusion

This study demonstrates that attitudes toward the CC in the context of EC remain divided within the Polish pharmacy community, reflecting broader ethical, legal, and sociopolitical tensions surrounding reproductive healthcare. Professional uncertainty appears to arise not only from personal beliefs but also from the lack of clear and consistent regulatory frameworks governing pharmacists’ responsibilities and rights in ethically sensitive situations.

From a policy perspective, the findings suggest a need for clear national guidelines defining the scope, procedures, and documentation requirements related to conscientious objection in pharmacy practice. We recommend that such guidelines specify 1) the exact circumstances under which pharmacists may invoke the CC, 2) required documentation and notification procedures, and 3) protocols to ensure timely patient access to EC when a pharmacist objects. Such regulations could help reduce legal ambiguity, support pharmacists in ethically complex situations, and ensure consistent access to time-sensitive services such as EC.

From a practical standpoint, strengthening professional training in reproductive health and legal aspects of pharmaceutical services may further improve decision-making, counselling quality, and patient access. Establishing transparent procedures that reconcile professional conscience with patient rights may therefore represent a key step toward improving both regulatory clarity and equitable healthcare provision.

## Data Availability

The original contributions presented in the study are included in the article/supplementary material, further inquiries can be directed to the corresponding author.
